# Two-Fold *ND5* Genes, Three-Fold Control Regions, lncRNA, and the “Missing” *ATP8* Found in the Mitogenomes of *Polypedates megacephalus* (Rhacophridae: *Polypedates*)

**DOI:** 10.3390/ani13182857

**Published:** 2023-09-08

**Authors:** Ling-Na Cai, Li-Hua Zhang, Yi-Jie Lin, Jing-Yan Wang, Kenneth B. Storey, Jia-Yong Zhang, Dan-Na Yu

**Affiliations:** 1 College of Life Sciences, Zhejiang Normal University, Jinhua 321004, China; cailingna@zjnu.edu.cn (L.-N.C.); 945496914@zjnu.edu.cn (Y.-J.L.); wangjingyan@zjnu.edu.cn (J.-Y.W.);; 2 Taishun County Forestry Bureau, Wenzhou 325200, China; zlh8701@126.com; 3 Department of Biology, Carleton University, Ottawa, ON K1S 5B6, Canada; kenstorey@cunet.carleton.ca; 4 Key Lab of Wildlife Biotechnology, Conservation and Utilization of Zhejiang Province, Zhejiang Normal University, Jinhua 321004, China

**Keywords:** *Polypedates megacephalus*, *ATP8* gene, mitogenome, long non-coding RNA (lncRNA), mitochondrial gene expression

## Abstract

**Simple Summary:**

The spot-legged treefrog *Polypedates megacephalus* (Anura: Rhacophoridae) is widely distributed in Asia. In previous studies, it has been noted that the mitochondrial gene *ATP8* has not been confidently annotated in any sequenced *Polypedates* to date. Duplications of control regions (CRs) are often observed in the tree frog family Rhacophoridae, and in most cases the copied CRs are highly similar to one another. This feature may lead to sequence assembly errors. Our findings are the first to detect two copies of the *ND5* genes and three copies of the CRs in *P. megacephalus* by employing a “primer bridging” approach and allude to the presence of *ATP8* via bioinformatic analyses and RT-qPCR. However, the question of whether the *ATP8* is functional needs to be addressed in future studies.

**Abstract:**

In prior research on the mitochondrial genome (mitogenome) of *Polypedates megacephalus*, the one copy of *ND5* gene was translocated to the control region (CR) and the *ATP8* gene was not found. Gene loss is uncommon among vertebrates. However, in this study, we resequenced the mitogenomes of *P. megacephalus* from different regions using a “primer bridging” approach with Sanger sequencing technologies, which revealed the “missing” *ATP8* gene in *P. megacephalus* as well as three other previously published *Polypedates*. The mitogenome of this species was found to contain two copies of the *ND5* genes and three copies of the control regions. Furthermore, multiple tandem repeats were identified in the control regions. Notably, we observed that there was no correlation between genetic divergence and geographic distance. However, using the mitogenome, gene expression analysis was performed via RT-qPCR of liver samples and it was thus determined that *COIII*, *ND2*, *ND4*, and *ND6* were reduced to 0.64 ± 0.24, 0.55 ± 0.34, 0.44 ± 0.21 and 0.65 ± 0.17, respectively, under low-temperature stress (8 °C) as compared with controls (*p* < 0.05). Remarkably, the transcript of long non-coding RNA (lncRNA) between positions 8029 and 8612 decreased significantly with exposure to low-temperature stress (8 °C). Antisense *ND6* gene expression showed a downward trend, but this was not significant. These results reveal that modulations of protein-coding mitochondrial genes and lncRNAs of *P. megacephalus* play a crucial role in the molecular response to cold stress.

## 1. Introduction

The genus *Polypedates* originated primarily in the islands of Southeast Asia during the Oligocene period and currently comprises 26 species that are widely distributed in South Asia, Southeast Asia, and East Asia [1]. Climate fluctuations have had a significant impact on the distribution of *Polypedates* species. With the effect of global warming, *Polypedates* have spread from Sundaland to mainland Southeast Asia and East Asia [2]. Low temperatures are thought to be the primary factor affecting the northward dispersal of *Polypedates* on the Chinese mainland, where they are mostly distributed in regions south of the Tsinling Mountains. Mitochondria, known as the cell’s energy factories, generate ATP via oxidative phosphorylation [3]. The genome of mitochondria is independent of the nucleus. Additionally, mitochondria possess the ability to resist and adapt to the impact of cold stress in order to maintain energy balance. This response may differ for various levels, including gene expression [4,5], protein levels [6,7], and enzyme activity [7,8]. Numerous studies have demonstrated that mitochondrial genes alter their expression to adapt to adverse environmental conditions [9,10,11,12]. However, few studies have investigated the impact of hypothermic stress on mitochondrial gene expression in the *Polypedates* genus.

The mitogenomes of tree frogs are arranged compactly, comprising 37 genes that include 13 protein-coding genes (PCGs), 22 tRNAs, and two ribosomal RNAs [13,14,15]. The length of mitogenomes varies depending on the length and number of control regions (CRs), generally ranging from 16 to 22 kb. Gene rearrangement is a frequent phenomenon in tree frogs and can be explained by the tandem duplication–random loss (TDRL) model [16] and the slipped-strand mispairing model [17]. Among the known species of tree frogs, mitochondrial *ND5* gene rearrangements are classified into the following two types: (1) those in which the *ND5* gene is shifted from between *ND4* and *ND6* genes to between the CR and LTPF gene cluster (*Buergeria buergeri* [18], *Zhangixalus dennysi* [19]), and (2) those in which the *ND5* gene is translocated between the two CRs (*Zhangixalus schlegelii* [20], *P. impresus* [21], *Polypedates mutus* [21], *Polypedates braueri* [22]). The mitogenome of *P. megacephalus* was initially sequenced by Zhang et al., revealing a 16,473 bp genome lacking the *ND5* gene and the *ATP8* gene [23]. Later, Huang et al. revised the length of the mitogenome to 19,952 bp and discovered that the *ND5* gene had not been lost, but rather was shifted to between two CRs with high similarity [24]. The occurrence of duplicated CRs is common in the Neobatrachia suborder, not only in Rhacophoridae [20,22,24,25] but also in other families such as Mantellidae [26,27] and Dicroglossidae [28,29]. This pattern of duplicated CRs is phylogenetically conserved and may be attributed to independent [30,31] or concerted evolution [32,33,34].

The 13 PCGs are crucial for cellular ATP synthesis and play a major role in oxidative phosphorylation [3,35]. ATP synthase is a complex composed of multiple subunits encoded by mitochondrial and nuclear genes, which form the F0 and F1 functional domains. The *ATP8* gene encodes the A6L subunit of the F0 complex. This subunit has a conserved amino acid motif MPQL and N-terminal transmembrane helix that shows considerable homology with one of the two b subunits from α-proteobacteria, suggesting that A6L is an evolutionary remnant of this bacterial subunit [36,37]. Previous research has focused on the absence of the *ATP8* gene in invertebrates such as Mollusca [38,39], Rotifera [40,41], and Nematoda [42]. However, recent studies have detected the presence of the *ATP8* gene in flatworms via manual annotations and transcriptomic data [43]. Despite this, due to its short length and highly divergent nature even within a genus, the *ATP8* gene is not conserved in flatworms [44]. Gene loss is rare among vertebrates compared to gene rearrangement, but it has been reported that the *ATP8* gene is missing only in the genus *Polypedates* [21,23,24]. Whether the *ATP8* gene was actually “missing” in *Polypedates* has become a concern for researchers.

Herein, we re-sequenced and re-annotated the mitogenomes of *P. megacephalus* from eight different locations. Surprisingly, a novel rearrangement with twofold *ND5* genes and threefold CRs was found for the first time in anurans. Furthermore, via manual annotation and RT-qPCR, we detected a novel *ATP8* located upstream of *ATP6*, and a lncRNA located between *ATP8* and *tRNA^Lys^*. In addition, we analyzed the expression of 13 mitochondrial PCGs and two lncRNAs in the liver under low-temperature stress to gain a deeper understanding of the molecular mechanisms in *P. megacephalus* under cold stress.

## 2. Materials and Methods

### 2.1. Sampling and Treatments

To obtain the subjects, 20 male *P. megacephalus* were captured from a rice paddy in Conghua, Guangdong Province, China on 21 June 2021. All animals were fed yellow mealworms in a plastic box at 25 °C for one week. From this group, 10 randomly selected frogs were transferred to a plastic box and placed at 8 °C for 24 h as the stress group. Meanwhile, another 10 frogs were kept in another plastic box at a constant temperature of 25 °C for 24 h to serve as a control group. All frogs were euthanized via double pithing, and their livers were removed and placed into pre-chilled cryotubes before being immediately flash-frozen in liquid nitrogen. Liver tissue samples were subsequently stored at −80 °C for downstream molecular analysis. We also obtained samples of *P. megacephalus* from Jinhua, Zhejiang, China (ZJJH); Fuqing, Fujian, China (FJFQ); Guilin, Guangxi, China (GXGL); Qinyuan Guangdong, China (GDQY); Huidong, Guangdong, China (GDHD); Wenzhou, Zhejiang, China (ZJWZ); and Phuket, Thailand (TGPJ) from 2010 to 2022, which were used to acquire the complete mitogenomes (Table S1).

### 2.2. DNA Extraction and Sequencing

**Figure 1:**
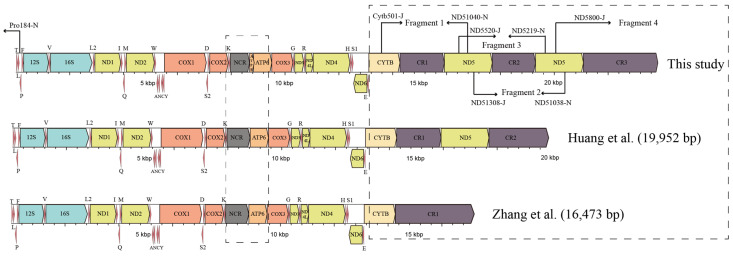
Illustration of sequencing strategy for mtDNA in *P. megacephalus.* Fragments 1–4 represent the four amplified segments. Primer names are shown above or below the arrows. The differences between this study and prior research (Zhang et al. [23], Huang et al. [24]) are emphasized with the dashed boxes.

Total genomic DNA was extracted from a clipped toe using the Ezup Column Animal Genomic DNA purification Kit (Sangon Biotech Company, Shanghai, China) in compliance with the manufacturer’s standard protocols for tissue. We amplified 10 overlapping genes via normal PCR and long-accurate PCR methods slightly modified from Zhang et al. [45]. Additionally, the *ND5* fragments were successfully obtained using primers designed by Cai et al. [46]. After obtaining the *ND5* gene fragment, four sets of primers were designed to sequence adjacent gene fragments, including the region from *ND5*_(1)_ to *ND5*_(2)_ genes, the region from *Cytb* to *ND5*_(1)_ genes, and the region from *ND5*_(2)_ to *tRNA^Pro^* genes (Figure 1). An overview of all fragments is given in Table 1 and Table S2. Unexpectedly, the amplicons generated from *Cytb* to *ND5*_(1)_, *ND5*_(1)_ to *ND5*_(2)_*,* and *ND5*_(2)_ to *tRNA^Pro^* fragments were found to have lengths of approximately 3400 bp, 3300 bp, and 4100 bp, respectively. These PCR products underwent bidirectional sequencing utilizing the primer-walking method from Sangon Biotech Company (Shanghai, China).

### 2.3. Assembly and Annotation

Via the DNASTAR Package v.7.1 [47], all mitogenomes were assembled sucessfully. Meanwhile, the tRNAscan-SE 2.0 web server (http://mitos.bioinf.uni-leipzig.de/index.py, accessed on 5 December 2022) [48] was used to identify the tRNA genes. Referring to the mitogenomes of *P. megacephalus* published in the GenBank database (AY458598, MH936677), two rRNAs and 12 PCGs (excluding the *ATP8* gene) were identified via MEGA 7.0 [49]. To analyze the *ATP8* sequences, we utilized SMART [50] with default parameters to identify domains. The hydrophobicity profiles of the resulting amino acid sequences were generated via the ExPASy tool ProScale [51]. In addition, AlphaFold2 (https://colab.research.google.com/github/sokrypton/ColabFold/blob/main/AlphaFold2.ipynb, accessed on 19 April 2023) [52], in combination with the Swiss-Model server (SWISS-MODEL (expasy.org), accessed on 1 May 2023) [53], were also applied to predict the structure of the *ATP8* proteins. An identical approach was applied to locate *ATP8* in the previously published mitogenomes of *P. mutus*, *P. braueri*, and *P. impresus*. The AT% and relative synonymous codon usage (RSCU) of five complete genomes were calculated using MEGA 7.0 [49]. Tandem Repeats Finder (http://tandem.bu.edu/trf/trf.submit.options.ht-ml, accessed on 5 January 2023) [54] was utilized to identify tandem repeats. The GC-skew and AT-skew indices were calculated with the following equations: GC-skew = (G − C)/(G + C), AT-skew = (A − T)/(A + T).

### 2.4. Molecular Phylogenetic Analyses

A total of 19 species mitogenomes of Rhacophoridae, including five newly complete (GDCH, GXGL, ZJJH, FJFQ, TGPJ) and three almost-complete (GDHD, GDQY, ZJWZ) mitogenomes of *P. megacephalus*, were used for phylogenetic analyses (Table S3). *Mantella madagascariensis* (GenBank: AB212225) and *Mantella baroni* (GenBank: MH141579) were used as outgroups. Due to the sequence divergence in duplicate *ND5* in TGPJ and FJFQ and the high heterogeneity of *ATP8* in the *Polypedates*, 11 PCG genes and two rRNA genes were aligned using MAFFT v 7.475 [55]. Using Phylosuite v1.2.3pre3 [56], all genes were extracted, Gblocked, and concatenated into a line under general default parameters and workflow. Ultimately, these 13 processed genes consisted of a mitogenome dataset with a length of 11,406 bp. Then, the dataset’s optimal partitioning scheme and specific nucleotide substitution models were determined via PartitionFinder v2.1.1 [57] with general default parameters. The specific partition schemes and the corresponding best-fit models selected for each part are documented in detail in Table S4. The GTR+I+G model was selected for maximum likelihood (ML) and Bayesian inference (BI) analyses. The ML analysis was performed with RaxML 8.2.0 [58] with rapid inference based on 1000 ultrafast repetitions. Meanwhile, the BI analysis was performed with MrBayes 3.2 over a total of 10 million generations, and the mean standard deviation of Bayesian split frequencies was below 0.01. The first quarter of generations were removed as burn-in. Based on the Kimura 2-parameter (K2P) model, the genetic distances between different geographic sites were calculated separately. Additionally, geographical distance was calculated from longitude and latitude. The strength of correlations between the genetic distances and the geographical distance matrices was tested using Mantel tests in GenALEx v6.0 [59].

### 2.5. RT-qPCR

Total RNA was extracted from liver samples of the control and 24 h cold-exposed (at 8 °C) *P. megacephalus* GDCH using a TakaRa MiniBEST Universak RNA Extraction Kit (Takara, Japan), according to manufacturer’s instructions. Quality control and reverse transcription of the extracted RNA were conducted following the procedures established by Jin et al. [10]. According to the newly obtained sequence of *P. megacephalus* from Conghua, Guangdong, Primer Premier 5.0 [60] was used to design specific primers for reverse transcription-quantitative polymerase chain reaction (RT-qPCR) (Table 2). The amplification products ranged from 97 to 155 bp in length, with melting temperatures between 48 °C and 50 °C and primer lengths between 18 and 22 bp. Three technical replicates were used to assess the genes corresponding to each primer pair. RT-qPCR was then performed using the conditions specified by Jin et al. [10].

The data are presented as the mean expression levels (±SE) for each experimental condition based on four independent experimental replicates from different individuals. Grubbs’ test was applied to eliminate outliers. Statistical assessment of mRNA expression was conducted using a *t*-test, with significance set at *p* < 0.05 as compared to the control group.

## 3. Results

### 3.1. Genome Organization and Gene Arrangement

In the present study, we obtained five complete mitogenomes and three partial mitogenomes of *P. megacephalus* from diverse geographic zones (Table S5). The newly sequenced complete mitogenomes varied in size from 23,798 bp in FJFQ to 24,103 bp in ZJJH. All mitogenomes contained 13 identified open reading frames (including the extra copy of the *ND5*), two rRNA genes, 22 transfer RNA genes, three CRs, and one long non-coding region (LNCR). Additionally, a short form of *ATP8* with an ATG start codon and a TAG stop codon was identified, which were previously unannotated in *P. megacephalus*. As previously reported in other anurans, the majority of the genes were coded on the H-strand, with the exception of *ND6* and eight tRNA genes (*tRNA^Pro^*, *tRNA^Gln^*, *tRNA^Ala^*, *tRNA^Asn^*, *tRNA^Cys^*, *tRNA^Tyr^*, *tRNA^Ser^*, and *tRNA^Glu^*) (Table S6). The RSCU of five complete mitogenomes was identical to that of previous studies [24] (Figure S1).

**Figure 2:**
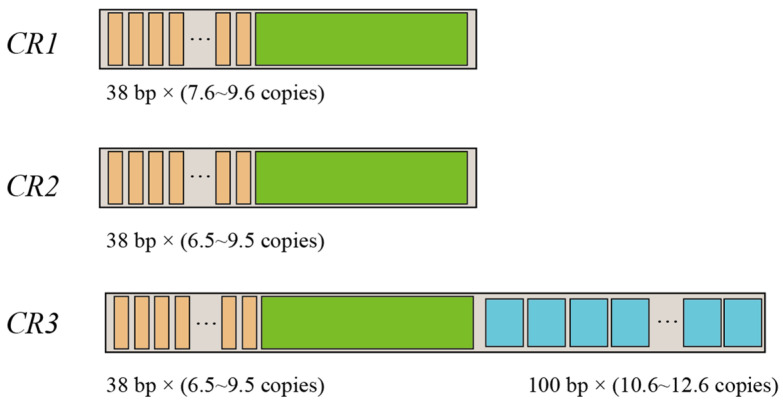
Characteristics of *P. megacephalus* CRs. The number of copies of tandem repeats within each CR varied between geographic sites. The orange squares are tandem repeat units 38 bp in length. The sky-blue squares are 100 bp-long tandem repeat units (only in the third control region). The green squares represent conserved sequences common to the three control regions.

All mitogenomes of *P. megacephalus* in this study contained threefold CRs and twofold *ND5* genes. We named these control regions *CR1* (between the *Cytb* and *ND5*_(1)_ genes), *CR2* (between the *ND5*_(1)_ and *ND5*_(2)_ genes), and *CR3* (between the *ND5*_(2)_ and *tRNA^Thr^* genes). The polymorphism of CR length was determined from the variable sizes and copy numbers of tandem repeats, resulting in size variations of the mitogenomes of the five *P. megacephalus* examined in this study. As shown in Table S7 and Figure 2, among these mitogenomes, tandem repeat units of 38 bp were detected in the 5’ sides of *CR1*, *CR2*, and *CR3*, whereas only the 3’ side of *CR3* possessed tandem repeat units of 100 bp. Further comparison indicated that the 5’ side of three CRs contained nearly identical sequences (around 99% similarity). Three CRs also showed higher AT content (around 66.3% for both CR1 and CR2, and about 70.1% for CR3) than in the whole genome (around 61.4%). Specifically, two identical *ND5* genes were detected in the genome from frogs collected at the GDCH, GXGL, and ZJJH locations, whereas two similar *ND5* genes were found in the TGPJ and FJFQ groups (nearly 98.5% similar sequence). Furthermore, the length of *ND5* was 1779 bp except for TGPJ (1785 bp).

### 3.2. ATP8 Annotation

**Figure 3:**
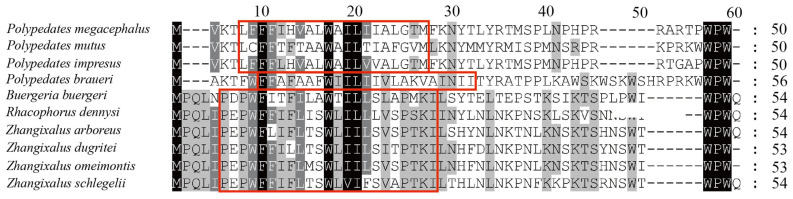
Amino acid alignment of *ATP8* in *Polypedates* and other Rhacophoridaes. The transmembrane regions are highlighted with red boxes.

Due to the annotation challenge presented by its short length and high variability, *ATP8* could not be reliably annotated via automated tools in the assembled mitogenomes. Hence, to identify this gene, a series of manual curation steps were performed. The newly annotated *ATP8*, which was located upstream of and shared four bases (ATAG) with *ATP6*, had low amino acid similarity to other frogs (similarity < 30%). Among these mitogenomes, the putative *ATP8* was significantly shorter than its counterparts in other Rhacophoridaes except for *P. bruari*. In all but one *Polypedates*, the starting amino acid sequence is MVKT, whereas in *P. bruari*, it was MAKT. As for the four *Polypedates* species, the *ATP8* gene similarity was determined to be 30%, whereas there was 86% similarity between *P. impresus* and *P. megacephalus.* Despite lowering the stringency requirements, BLASTN and BLASTX failed to detect the ORF of the *ATP8* gene. By using SMART, we found a transmembrane domain of only about 20 amino acids in the putative *ATP8* sequence of the genus *Polypedates* (Figure 3), whereas no signal peptide or ATPase domain was found.

**Figure 4:**
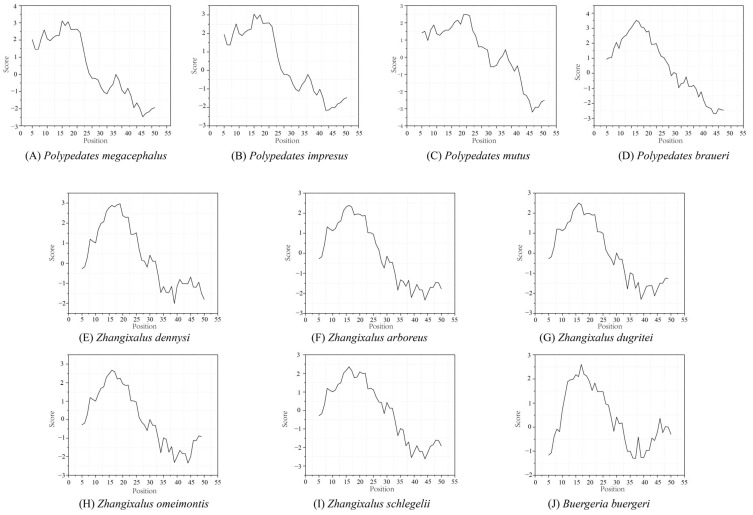
The hydrophobicity profiles of the putative *ATP8* amino acid sequences of *Polypedates* and other reported Rhacophoridae species.

For comparison, the hydrophobic patterns of the annotated *ATP8* in the Rhacophoridae family are depicted in Figure 4. The hydrophobicity profiles of the putative *ATP8* amino acid sequences of *Polypedates* and other reported Rhacophoridae species showed remarkably similar graphs, with positive hydrophobicity at the N-terminus and largely negative scores at the C-terminus. ATP synthase protein 8 was detected using Swiss-model, albeit with low support (*P. megacephalus*: 10.71% sequence identity; *P. mutus*: 28.57% sequence identity; *P. impresus*: 16% sequence identity; *P. braueri*: 20% sequence identity). In addition, based on the amino acid sequence alone, the three-dimensional (3D) structure of the *ATP8* protein was predicted within Alphafold2 [52]. As depicted in Figure S2, the *ATP8* domain structures of all 10 Rhacophoridae species were accurately projected, whereas the precision and confidence of the C-terminus was found to be relatively low.

**Figure 5:**
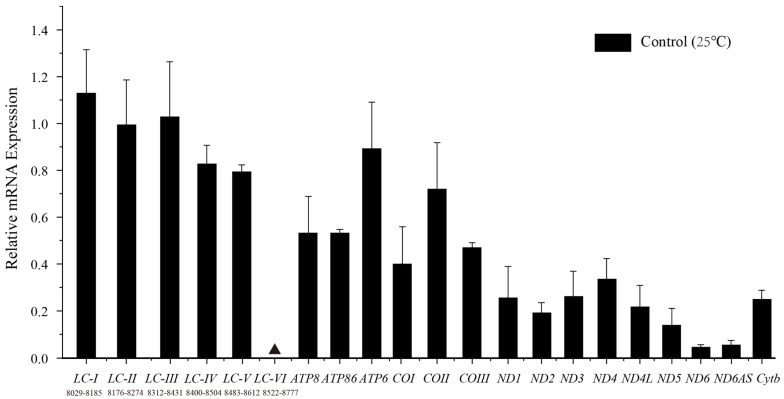
Relative mRNA expression levels of genes encoding mitochondrial proteins as well as two lnc RNAs at 25 °C. The solid black triangle represents undetected gene expression. The numbers under *LC-I* to *LC-VI* are amplified fragment ranges. Relative mRNA levels were determined via RT-qPCR with *n* = four independent biological replicates and outliers within the group were removed via Grubbs’ method: the detection level α was 0.05, the critical value GP(*n*) was 1.46, and outliers were removed when the calculated value Gi > GP(*n*). Results are expressed as mean ± SE. Relative transcript levels were standardized using β-actin transcript levels as the reference gene.

Confirmation of transcription of the idiosyncratic *ATP8* was achieved via successful amplification of an RT-PCR product from the predicted partial *ATP8,* using total RNA isolated from GDCH as the template. To further ensure the accuracy of *ATP8* annotation, a pair of quantitative primers (*ATP86*) spanning *ATP8* and *ATP6* were designed, and gene expression in this region was detected. The results indicated no significant differences in expression levels between *ATP8* and *ATP6* (Figure 5).

### 3.3. Long Non-Coding RNA

In *P. megacephalus*, an LNCR with high AT% (58.0%) between *tRNA^Lys^* and *ATP8* genes was detected. The length of the LNCR was 713 bp except in *P. megacephalus* GXGL (708 bp). In line with previous studies, this region included a fragment with high similarity to the *tRNA^Lys^* gene (around 76%) that failed to fold into a typical cloverleaf secondary structure. To confirm whether the LNCR can be transcribed into RNA, six pairs of quantitative primers were designed to measure the expression of this region. Among them, the expression levels of the products amplified by the first five primer pairs (LC-I, LC-II, LC-III, LC-IV, LC-V) were significantly (1.8–20.4 times) higher than those of protein-coding genes (except for *ATP6* and *COII*, which showed no significant difference) (Figure 5). By contrast, no gene expression was detected in the sixth segment (LC-VI). Due to the difficulty in designing quantitative primers, a 38 bp gap existed between the first amplified segment and the second amplified segment. Nevertheless, as there was no difference in expression levels among the first five segments, we concluded that the first five primer pairs measured gene expression at different positions on the same RNA transcript. Furthermore, the abundance of the antisense *ND6* transcript (*ND6AS*) was higher than that of the sense *ND6* transcript (*p* > 0.05).

### 3.4. Gene Expression of P. megacephalus under Low-Temperature Stress

**Figure 6:**
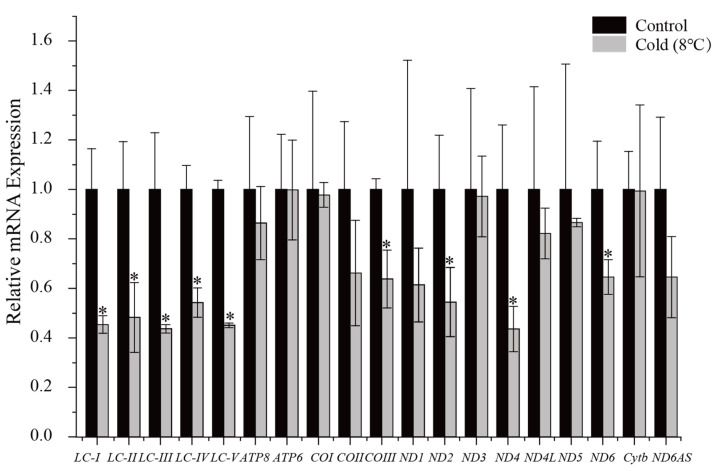
Relative mRNA expression levels in liver before and after low-temperature stress at 8 °C in *P. megacephalus*. Mean values for controls were set to 1.0, and values for anoxic frogs are expressed relative to controls. Statistical significance was assessed with a two-tailed Student’s *t*-test, where * denotes a significant difference from the corresponding control; *p* < 0.05. Other information as in Figure 5.

Under hypothermic stress (8 °C for 24 h), the transcriptional levels of the 13 mitochondrial PCGs from *P*. *megacephalus* GDCH liver were measured to compare control at 25 °C with 24 h cold exposure. Transcriptional levels of *COIII*, *ND2*, *ND4*, and *ND6* were reduced to 0.64 ± 0.24, 0.55 ± 0.34, 0.44 ± 0.21 and 0.65 ± 0.17, respectively, as compared with controls (*p* < 0.05). The relative transcriptional levels of the remaining nine protein-coding mitochondrial genes, as well as antisense *ND6*, did not change in response to cold exposure. In addition, we determined that the transcriptional levels of the long noncoding region between *tRNA^Lys^* and *ATP8* (LC-I, LC-II, LC-III, LC-IV, LC-V) were significantly reduced to values of 0.45 ± 0.075, 0.48 ± 0.28, 0.44 ± 0.04, 0.54 ± 0.12, 0.45 ± 0.02, respectively, as compared to controls (*p* < 0.05). Furthermore, there was no statistically significant difference in the transcript abundance of these five regions (Figure 6).

### 3.5. The Genetic Distances and Phylogenetic Relationships of Polypedates

The interspecific genetic distance within *P*. *megacephalus* was at least 0.001, ranging from 0.001 to 0.058. GDQY and GDCH showed the shortest geographical distance between all sampling locations, whereas TGPJ and ZJJH were the farthest apart (Table S8). Additional Mantel tests of isolation by distance demonstrated a statistically non-significant correlation between geographic and genetic distances (*R*^2^ = 0.096, *p* = 0.08) (Figure S3).

**Figure 7:**
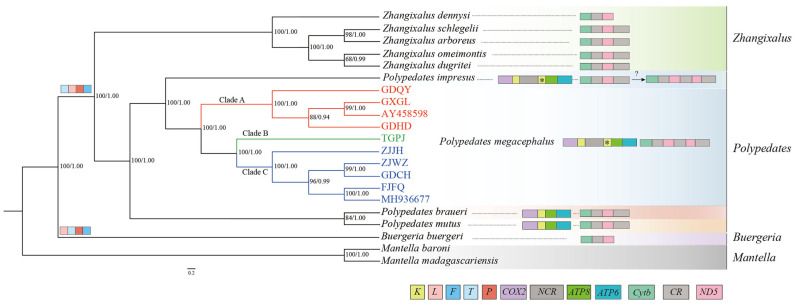
Phylogenetic tree constructed based on the nucleotide dataset of 11 PCGs and two rRNAs using BI and ML methods. *Mantella baroni* and *Mantella madagascariensis* were used as the outgroups. The numbers on branches display posterior probabilities (PP)and bootstrap support (BS) as determined from BI (**right**) and ML (**left**), respectively. Box images on the right show gene rearrangements for the Rhacophoridae species involved in this study. Different colored boxes represent different genes. Asterisked genes indicate pseudogenes.

The results from the two phylogenetic analyses (BI and ML) of the 11 protein-coding genes and two rRNAs showed similar topologies, supporting the previous classification (Figure 7). The phylogenetic analyses of nucleotide datasets with high bootstrap values revealed the formation of two independent sister groups: one composed of *P. mutus* and *P. braueri* and the other composed of *P. megacephalus* and *P. impresus*. All specimens of *P. megacephalus* collected from various localities formed a single clade, which was further divided into three subclades (Clade A, Clade B, and Clade C) (Figure 7). However, within Clade C, the frogs in GDCH did not cluster with those in GDHD and GDQY, despite their close geographic proximity. The two previously published *P. megacephalus* genomes (AY458598, MH936677) from Guangxi Province were assigned to Clade A and Clade C, respectively.

## 4. Discussion

### 4.1. Gene Rearrangements and Rearrangement Mechanisms

**Figure 8:**
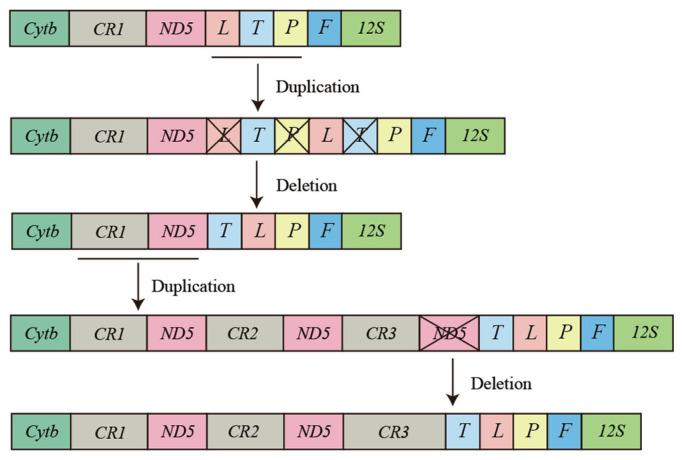
Proposed mechanism of gene rearrangements in *P. megacephalus*. Gene sizes are not drawn to scale. Different colored boxes represent different genes. Horizontal lines and crossed-out colored boxes represent gene duplications and gene deletions, respectively.

Vertebrate mitogenomes are typically compact and relatively conserved [15]. As the amount of whole mitogenome data for vertebrates has dramatically increased, gene rearrangement phenomena have become a common occurrence in the mitogenomes of vertebrates [61,62,63]. In addition, mitochondrial gene rearrangements are thought to result from tandem duplication of gene regions due to slipped-strand mispairing and the deletion of redundant genes [64,65]. The revised *P. megacephalus* mitogenomes possessing threefold control regions as well as twofold *ND5* genes and a pseudogene of *tRNA^Lys^* seem to prove the occurrence of duplication-and-deletion events. Notably, the existence of three control regions has also been reported in sand lizards [66], while tiger frogs have been found to possess twofold *ND5* genes [63]. However, the *Cytb-CR1-ND5*_(1)_*-CR2-ND5*_(2)_-*CR3*-*tRNA^Thr^-tRNA^Leu^-tRNA^Pro^-tRNA^Phe^* gene rearrangement was first reported in anurans. The genus *Buergeria* was considered to be the most basal group of the Rhacophoridae family with the “*Cytb*-*CR-ND5-tRNA^Leu^-tRNA^Thr^-tRNA^Pro^-tRNA^Phe^*” gene arrangement [18,67,68]. The TDRL model is the most valuable and commonly referenced model for understanding the diversity of gene rearrangements in vertebrate mitogenomes [16,69]. Therefore, we hypothesize that at least two duplication–deletion events must have occurred to explain the rearrangements of *P. megacephalus*. One rearrangement is a tandem duplication in the area encompassing the *CR* and *ND5* genes. The steps of the TDRL are as follows. First, the *CR-ND5* gene cluster was tandemly replicated three times and generated three sets of the identical gene cluster (*CR1*-*ND5*_(1)_-*CR2*-*ND5*_(2)_-*CR3*-*ND5*_(3)_). Secondly, the *ND5*_(3)_ gene was randomly eliminated, and the new order *CR1*-*ND5*_(1)_-*CR2*-*ND5*_(2)_-*CR3* was generated. The other tandem duplication event took place in the region harboring the *tRNA^Leu^*, *tRNA^Thr^*, *tRNA^Pro^*, and *tRNA^phe^* genes. The TDRL appears to comprise the following steps: the *tRNA^Leu^*-*tRNA^Thr^*-*tRNA*^Pro^ gene cluster underwent two tandem duplications, followed by elimination of *tRNA^Leu^*, *tRNA^Thr^*, and *tRNA^Pro^*, resulting in a new gene order of *tRNA^Thr^-tRNA^Leu^-tRNA^Pro^-tRNA^Phe^* (Figure 8).

### 4.2. Possible Causes for Misdiagnosis of a “Single” ND5 Gene

In a prior study, Huang [24] corrected the mitogenome of *P. megacephalus* using next-generation sequencing and LA-PCR and concluded that the *ND5* gene had translocated to the *CR* 3’ end instead of being lost. However, this conclusion contradicts the present study, which revealed the rearrangement structure of “*CR1*-*ND5*_(1)_-*CR2*-*ND5*_(2)_-*CR3*” using the “primer bridging” method. Re-analyzing Huang’s sequencing method and sequence characteristics revealed two factors that account for the discrepancy between Huang’s result and the present study: (1) In our study, LA-PCR amplification and Sanger sequencing with one set of primers (primers upstream and downstream were located on *ND5*) and resulted in a product with 3300 bp, suggesting the existence of two *ND5* genes. In Huang’s study, the *ND5* gene was obtained using two primer pairs: (a) (upstream primer 1: CytbFow1, downstream primer 1: FND512800H) and (b) upstream primer 2: ND5F2, downstream primer 2: R16M1). The first primer pair amplified the sequence of the first *ND5* gene, while the second primer pair amplified the sequence of the second *ND5* gene. (2) In addition, it was found that the three CRs in the mitogenomes exhibited highly similar sequences in the 5’ region, which could lead to sequence assembly errors (i.e., *CR2* and *CR3* were misassembled or combined (as one CR).

### 4.3. Long Non-Coding RNA

Long non-coding RNAs (lncRNAs) are generally defined as those that are >200 nucleotides, lack protein-coding capacity, and show great structural complexity and plasticity [70]. Most of them are generated by the nuclear genome. However, increasing evidence indicates that some lncRNAs are generated from the mitochondrial genome or located in mitochondria [71]. In our study, an abundant mitochondrial lncRNA was identified between the genes *tRNA^Lys^* and *ATP8* in GDCH, as detected via RT-qPCR. The lncRNA was punctuated by multiple stop codons, and no prominent ORFs were detected using verterabate mitochondrial code. The longest ORF spanned merely 129 amino acids without homologous sequences based on vertebrate codons. Our study also measured the expression levels of the lncRNA and compared them with transcripts of the 13 mitochondrial protein coding genes. What surprised us was that lncRNA transcriptional levels were significantly higher than those of the PCGs. Similarly, the lncRNA upstream of *ATP8* was also strongly expressed in *Stenostomum sthenum* [43]. Earlier research revealed that antisense transcripts of *ND5*, *ND6*, and *Cytb* genes play an essential role in maintaining mitochondrial mRNA stability or regulating gene expression via the formation of ribonuclease-resistant double-stranded structures with their respective complementary mRNAs [72]. Expression of an antisense *ND6* was also detected in our study. However, until now it was doubtful whether numerous lncRNAs have functions. This led us to think about what role a highly expressed lncRNA could play in the cell, how these mitochondrial lncRNAs could affect energy metabolism, and whether they could respond to injury and stress. These are the fundamental research questions that will receive attention in the future.

### 4.4. Reviving the Lost ATP8 Gene in Polypedates

The absence of the *ATP8* gene in *Polypedates* has garnered substantial interest among molecular systematists specializing in metazoans. It is worth noting that the *ATP8* protein has unique features, such as its variable and short length, and higher preservation of secondary structure compared to the primary sequence [73]. Both these features can render the annotation of *ATP8* more challenging in certain scenarios. Conventional tools failed to detect the *ATP8* genes in *Polypedates*. Therefore, the significance of manual annotation is emphasized.

The *ATP8* gene is one of the components of ATP synthase (F1F0), which contributes significantly to the electron transport chain, and deletion of the *ATP8* gene will prevent the electron transport chain from providing ATP to the organism and prevent the frog from reproducing. However, in reality, *Polypedates* are widely distributed and not naturally extinct. As automated gene annotation with MITOS failed to identify *ATP8* in either *P*. *megacephalus* or the other three published *Polypedates* mitochondrial genomes, we manually searched for *ATP8* candidates in all four mitogenomes. From this search, we managed to identify *ATP8* in *P*. *megacephalus* in the present study as well as all three published *Polypedates* mitogenomes. The presence of *ATP8* in *P*. *megacephalus* was supported by RT-qPCR, a transmembrane region at the N-terminus, and hydrophobicity profiles. These findings suggest that *ATP8* is not absent in all *Polypedates*. The phenomenon of *ATP8* gene loss is also common in flatworms, but recently, some researchers have used bioinformatics to demonstrate the existence of *ATP8* in some flatworm species: *Stenostomum leucops* [74], *S. sthenum* [43], and *Macrostomum lignano* [43]. In addition, Lubośny, Przyłucka, and Śmietanka [75] have confirmed the presence of *ATP8* in *Mytilus edulis* via proteomic approaches and manual curation procedures. In mammals, fish, and yeast, *ATP6* and *ATP8* are encoded in overlapping genes in mitochondrial DNA [76,77,78]. Additionally, it has been proven that the synthesis of *ATP6* is dependent on the translation of *ATP8* and occurs in association with a single ribopeptide transcript [79]. Among our resequenced sequences of *P. megacephalus*, these two genes shared a characteristic: a 4-nucleotide overlap of their sequences. Our quantitative data also demonstrated that *ATP6* and *ATP8* are contiguous transcripts.

In almost all animal lineages, there is strong selective pressure to maintain a minimal set of 37 genes [80]. It is imperative for researchers to exercise caution when regarding the absence of a mitochondrial gene [81]. Considering the traits of the *ATP8* gene, it is not unexpected that many organisms have been hypothesized to have undergone *ATP8* loss. This prompts the query of whether these organisms genuinely lack *ATP8*, or if this is merely a matter of annotation. Subsequent investigations into the functional roles and transcriptional activity of *ATP8* may prove to be essential. Unambiguous resolution of uncertainties surrounding “uncertain” protein-coding genes can only be achieved through experimental proteomic methods.

### 4.5. Transcriptional Levels of Mitochondrial Genes

In the present study, we found that metabolic depression of *P*. *megacephalus* was reflected at the transcriptional levels of the mitogenome in liver. The transcriptional level of the *COIII* gene in *P. megacephalus* was found to be significantly reduced in response to cold exposure (Figure 6). This finding is consistent with an earlier study that found that overwintering *Nanorana parkeri* frogs decreased mRNA expression of cytochrome oxidase, thereby reducing mitochondrial aerobic capacity and increasing survival time [82]. Additionally, a decrease in *COI* expression has also been observed in *Dryophytes versicolor* [11]. Cytochrome c oxidase (COX) serves as the terminal electron acceptor of the respiratory chain, facilitating the transfer of electrons to reduced oxygen to form water, and also exhibits proton-pumping activity [83,84]. Furthermore, defects in the assembly and function of COX can affect organs with high energy demands [85]. In a low-temperature environment, the decrease in COX activity and other mitochondrial enzymes in *P. megacephalus* may result from inhibited protein synthesis, which slows down mitochondrial metabolism. During cold stress, gene transcriptional levels of *ND2*, *ND4*, and *ND6* in liver tissues were significantly reduced in *P*. *megacephalus*. The observed suppression of the four NADH dehydrogenase subunits of *P*. *megacephalus* may represent a regulatory response to counteract the metabolic disturbances that occur in response to low temperatures. Furthermore, the lncRNA was down-regulated significantly in exposure to cold conditions, whereas antisense *ND6* gene expression showed a downward trend but this was not significant. These results indicate that the lncRNA is involved in adapting to fluctuating environmental temperatures. However, the precise molecular mechanisms that regulate this process remain unclear.

### 4.6. Associations between Phylogenetic Relationships and Gene Rearrangements

In the current study, the phylogenetic tree showed the pairs of *P. mutus* and *P. braueri* versus *P. megacephalus* and *P. impresus* formed separate decisive sister groups. This topology was consistent with that published previously in other studies [21,86,87]. Past studies revealed that the *P. leucomystax* complex was considered to be composed of seven highly supported clades [2,87,88]. The phylogenetic analyses conducted by Yuan [2] based on partial mitochondrial and nuclear genes indicated that a clade comprising three species, *P. megacephalus*, *P. leucomystax*, and *P. teraiensis*, formed a sister group to *P. impresus*, whereas *P. macrotis* and *P. mutus* formed a clade as sisters, which was inconsistent with our results. This difference was due to the fact that different datasets were used to reconstruct the phylogenetic relationship.

During natural dispersal, amphibians face multiple distance-related and biogeographic barriers. Brown proposed that the transportation of agricultural products facilitated the range expansion of *P. leucomystax* into the Philippines [89]. Phylogenetic analysis revealed that the GDCH frog did not cluster with GDHD and GDQY, which were geographically closer, but instead grouped with ZJWZ, which was further away. We speculate that this may be due to human transportation, leading to the establishment of a new geographical population, which is consistent with Brown’s perspective [89]. To enhance the comprehension of the taxonomic relationships among *Polypedates* species, additional molecular information is required to construct a fully accurate phylogenetic tree.

In our present study, the phylogenetic relationship within *Polypedates* offers highly convincing results that align with the gene arrangements and the NCR locations (Figure 7). Regarding the four *Polypedates* species analyzed in this study, it was observed that the genus was split into two distinct branches. One of these branches contained an LNCR between pseudogene *tRNA^Lys^* and *ATP8*. Based on the phylogenetic tree analysis, it was observed that two mitochondrial genomes with incomplete sequences (GDQY and GDHD) and the published mitochondrial genomes (AY458598) clustered with GXGL in Clade A. By contrast, ZJWZ clustered with three other mitochondrial genomes (ZJJH, GDCH, FJFQ) and the published mitochondrial genomes (MH936677) in Clade B. These results suggest that these species share the same “*CR1*-*ND5*_(1)_-*CR2*-*ND5*_(2)_-*CR3*” rearrangement structure. Additionally, *P. megacephalus* and *P. impresus* were sister taxa. The “*CR1*-*ND5*_(1)_-*CR2*-*ND5*_(2)_-*CR3*” rearrangement structure existed in *P. megacephalus*, whereas *P. impresus* exhibited a “*CR1-ND5-CR2*” structure. Therefore, further research is needed to determine whether *P. impresus* has the same rearrangement structure as the *P. megacephalus*.

## 5. Conclusions

In this study, we re-sequenced and re-annotated five complete and three almost complete mitogenomes of *P. megacephalus* from different geographic sites. A novel gene rearrangement of “*CR1*-*ND5*_(1)_-*CR2*-*ND5*_(2)_-*CR3*” was first detected in *P. megacephalus.* The gene rearrangement was explained via the TDRL model and the slipped-strand mispairing model. Based on the resemblance of protein tertiary structures (Figure S2), hydrophobic pattern similarities (Figure 4), and the results presented above, it is proposed that the *ATP8* gene could be present not only in *P*. *megacephalus* but also throughout the genus *Polypedates*. However, whether this *ATP8* gene can synthesize functional proteins remains to be further investigated in future studies. Analysis of mitochondrial gene expression in response to temperature change showed that the transcriptional levels of the *COIII*, *ND2*, *ND4*, and *ND6* were significantly reduced. Also, the lncRNA between positions 8029 and 8612 was transcribed at high levels and significantly down-regulated under low-temperature stress. These findings suggest that *P. megacephalus* alters mitochondrial gene expression to adapt to adverse environmental conditions.

## Figures and Tables

**Table 1:** PCR primer pairs, sequences, and estimated fragment length used in this study.

PCR Fragments	Primer Names	Primer Sequence (5′-3′)	Length of Fragments	Annealing Temperature
**F1**	Cytb501-J	GGCTTCTCAGTYGAYAATGC	~3400 bp	51 °C
	ND51040-N	TTYCGAATGTCYTGTTCATC		
**F2**	ND51308-J	MTCAACYATATCCGCTGTRR	~3300 bp	50 °C
	ND51038-N	YCGAATGTCYTGTTCATCAT		
**F3**	ND5520-J	TAGTAATCTTTTGCTGRGC	~3300 bp	50 °C
	ND5219-N	ATAGGAGGGAGTAGGTGTC		
**F4**	ND5800-J	TATGCTTAGGTGCTCTATCC	~4100 bp	50 °C
	Pro184-N	GAGTCAGTGGAAGAGGTTAA		

**Table 2:** RT-qPCR primers of *P. megacephalus* GDCH used in this study.

Gene Name	Forward Primers (5′-3′)	Reverse Primers (5′-3′)
*ND1*	GDBT-ND1-J1 TGCCCTTATTGGTTCTTTAC	GDBT-ND1-N1 AAGATGGACAGTGTGAAGCC
*ND2*	GDBT-ND2-J1 ACTGACCTCAACACACGC	GDBT-ND2-N1 GGTAAGAGGTGGGAGGC
*ND3*	GDBT-ND3-J1 TTCTGGCTGGCAACTCTG	GDBT-ND3-N1 TACAAGGAAGAAGCGTATGGA
*ND4*	GDBT-ND4-J1 GGGCTACGGCATCTTACG	GDBT-ND4-N1 GGCAGAGCAGGGCTGTTAT
*ND4L*	GDBT-ND4L-J1 GGCTCACCGAATACACTTACTT	GDBT-ND4L-N1 TGGGCAAAGGCTTAGGG
*ND5*	GDBT-ND5-J1 ACCGAATTGGAGACATTGGATT	GDBT-ND5-N1 GCAAGGATGAAGGCTATGAGAA
*ND6*	GDBT-ND6-J1 CGTCCAATCCGTCTCCGTT	GDBT-ND6-N1 GCAACCAGAGCTGAACAGTAAG
*ND6AS*	GDBT-ND6AS-J1 GCTGAACAGTAAGCAAACACA	GDBT-ND6AS-N1 TGGGTTTAGTGTGAGGTGC
*COI*	GDBT-COI-J1 CTACAAACTCCACGCTG	GDBT-COI-N1 GCGTCTGGGTAGTCTGAGTA
*COII*	GDBT-COII-J1 CAGGGCGGCTCACTCAA	GDBT-COII-N1 ATCGGTAGGGCTTCAAC
*COIII*	GDBT-COIII-J1 GGTCCTATTAGCCTCTGGG	GDBT-COIII-N1 TCGTAATACTCCATCGCTTG
*ATP6*	GDBT-ATP6-J1 ACAACCCAACTGCCACTAA	GDBT-ATP6-N1 GGGTGTGCCTTCTGGTAG
*Cytb*	GDBT-CYTB-J1 GACTGCTCCGTAATCTCCA	GDBT-CYTB-N1 AAATAGGAGAATAACACCGATG
*ATP8*	GDBT-ATP8-J1 CATTCACGTAGCCCTATGAGC	GDBT-ATP8-N1 GTGATATTGTGCGGTACAGTGT
ATP86	GDBT-ATP86-J1 ACACTGTACCGCACAATATCA	GDBT-ATP86-N1 GGGTTGGTGATGTAAATTGACT
LC-I	LC-I-J1 TTGCTTACTAAACTGCTGAG	LC-I-N1 GCGTATTATGATTCACAGGT
LC-II	LC-II-J1 CATAATACGCCCGTGGAC	LC-II-N1 AGAGCCGCACTCATTGGT
LC-III	LC-III-J1 AAGTGGCAAGTGCAACAATCA	LC-III-N1 GGCTCCGAGTGGATAAGAGG
LC-IV	LC-IV-J1 CCCACTTAATACCCTCTTATCC	LC-IV-N1 CGCTTGGTGAATAATCAGTTG
LC-V	LC-V-J1 GCAACTGATTATTCACCAAG	LC-V-N1 GGTGATTAGAGTTGTGGGAT
ACTIN	GDBT-ACTIN-J1 CATCAGGCAACTCGTAGC	GDBT-ACTIN-N1 GCGTGACATCAAGGAGAAG

## Data Availability

The data supporting the findings of this study are openly available from the National Center for Biotechnology Information at https://www.ncbi.nlm.nih.gov, accessed on 10 December 2022. Accession numbers are: OP965713-OP965718, OP936085-OP936086.

## Supplementary Materials

The following supporting information can be downloaded at: https://www.mdpi.com/article/10.3390/ani13182857/s1, Figure S1: Relative synonymous codon usage (RSCU) in five *P. megacephalus* mitogenomes; Figure S2: Three-dimensional(3D) structures of the *ATP8* protein of all 10 Rhacophoridae; Figure S3: Scatter plots of genetic distance vs. geographical distance for pairwise population comparisons; Table S1: Collection data of samples of *P. megacephalus*; Table S2: PCR primer pairs, sequences, and used in this study; Table S3: List of species used in the phylogenetic analyses; Table S4: The partition schemes and best-fitting models selected for the 11 protein-coding genes and two rRNA genes; Table S5: Base compositions of five *P. megacephalus* mitogenomes; Table S6: Locations of features in the mtDNA of *P. megacephalus* from five regions (ZJJH, FJBT, GDCH, TGPJ and GXGL); Table S7: Features of *P. megacephalus* control regions; Table S8: Pairwise genetic distance (below diagonal) and natural logarithm of geographical distance (km) (above diagonal) between geographical populations of *P. megacephalus* based on the mitogenomes.
