# Supplementary material for: Two-Fold ND5 Genes, Three-Fold Control Regions, lncRNA, and the “Missing” ATP8 Found in the Mitogenomes of Polypedates megacephalus (Rhacophridae: Polypedates)
Source: Animals (Basel). 2023 Sep 8;13(18):2857. doi: 10.3390/ani13182857 (PMC10525163; doi:10.3390/ani13182857)
Supplement: Supplementary file 1 [file animals-13-02857-s001.zip › animals-2486590-supplementary.pdf]

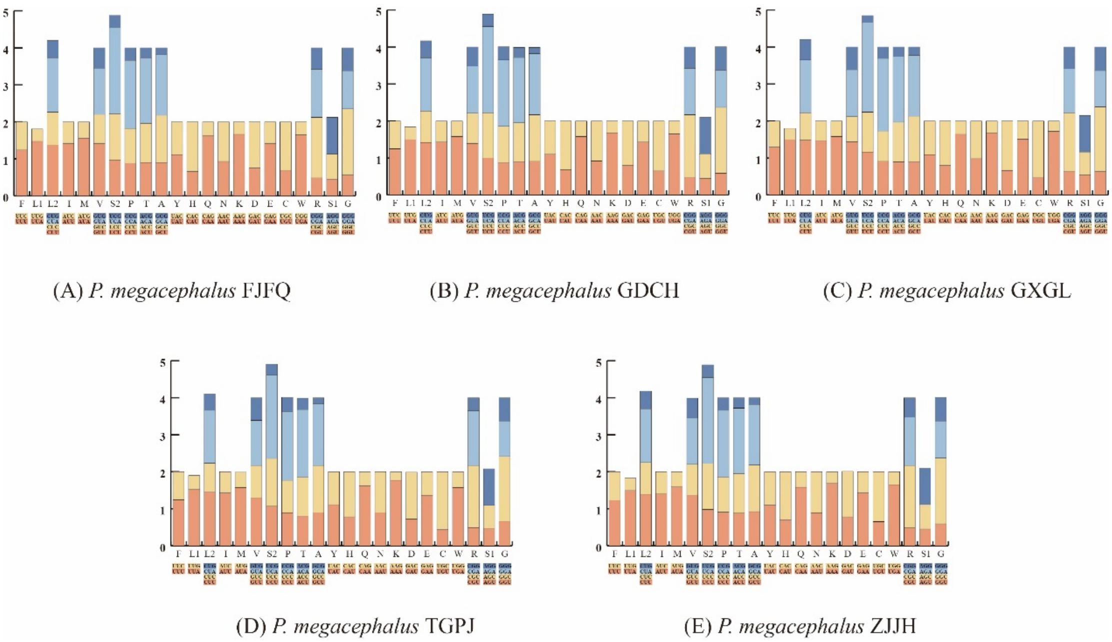

Figure S1. Relative synonymous codon usage (RSCU) in five *P. megacephalus* mitogenomes.

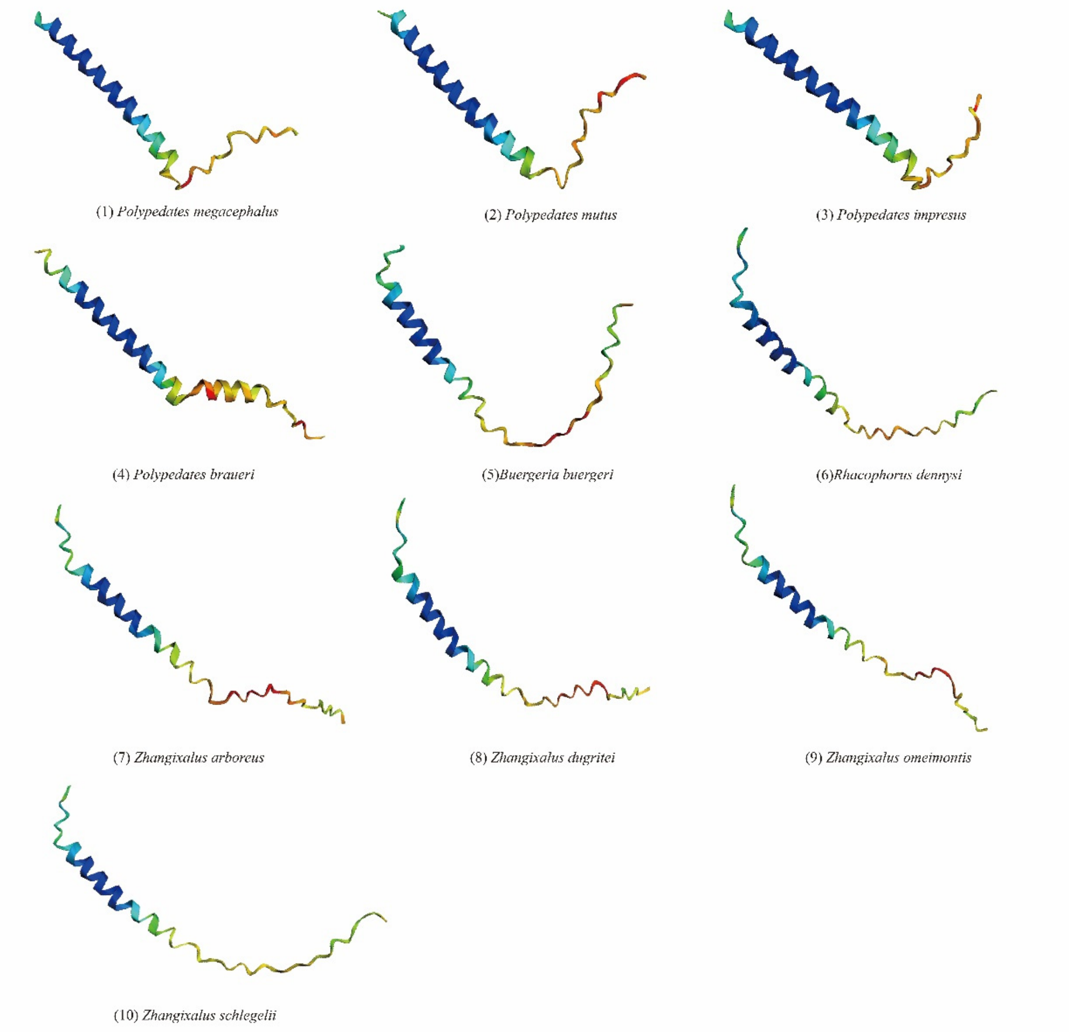

Figure S2. Three-dimensional(3D) structures of the *ATP8* protein of all 10 Rhacophoridae.

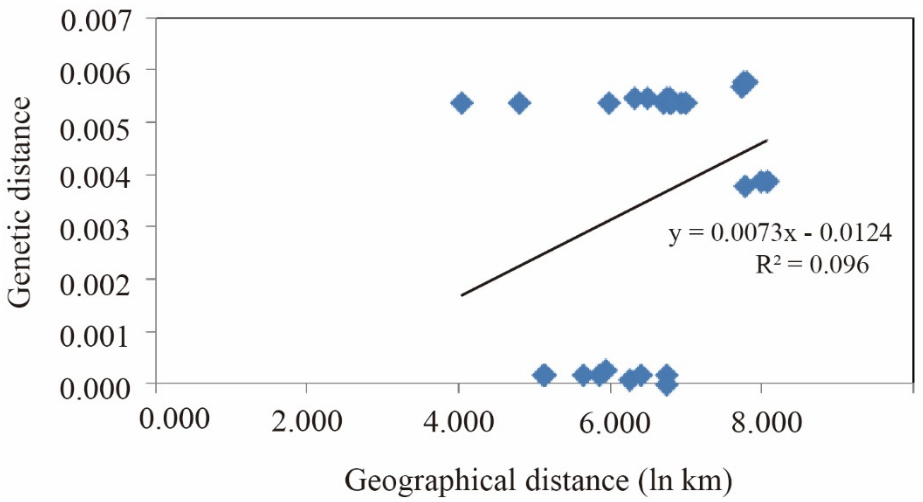

Figure S3. Scatter plots of genetic distance vs. geographical distance for pairwise population comparisons. The horizontal coordinate is Geographical distance (ln kilometer), and the vertical coordinate is Genetic distance.

Table S1. Collection data of samples of *P. megacephalus*.

**Samples**	**Collecting locality**	**Latitude**	**Longitude**
ZJJH	Wucheng, Jinhua, Zhejiang, China	29.14 °N	119.64 °E
ZJWZ	Chashan, Wenzhou, Zhejiang, China	27.92 °N	120.70 °E
GXGL	Xiangshan, Guilin, Guangxi, China	25.21 °N	110.25 °E
GDQY	Huadu, Guangzhou, Guangdong, China	23.58 °N	113.22 °E
GDCH	Conghua, Guangzhou, Guangdong, China	23.65 °N	113.78 °E
GDHD	Huidong, Huizhou, Guangdong,China	22.985 °N	114.71 °E
FJFQ	Fuqing, Fuzhou, Fujian, China	25.71 °N	119.25 °E
TGPJ	Phuket Island, Thailand	7.84 °N	98.37 °E

Table S2. PCR primer pairs, sequences, and used in this study.

PCR Fragments	Primer Names	**Primer Sequence (5' - 3')**	Length of Fragments
F5	P1-219-J	AAAGCATAGTGCTGAAAACGC	~600 bp
	P1-818-N	TTGTCGATTATAGAACAGGCTCCTCT	
F6	P2-1162-J	TAGAAGAGGCAAGTCGTAAC	~1,800 bp
	P2-2951-N	GGTGGGTGAGGTAAAGTATT	
F7	P3-2611-J	GGCTTACGACCTCGATGTTGGATCA	~1,400 bp
	P3-4062-N	GGTATGGGCCCAARAGCTT	
F8	P4-3917-J	GAAAGATAAGGAYCTCCTTGATAG	~1,400 bp
	P4-5347-N	AAGTAGAATGAAGCTCGCTGG	
F9	P5-5169-J	AGCCCGAAGGCCTTCAAAGC	~2,000 bp
	P5-7236-N	ATAATAGGGGATGCGGCGTCTTG	
F10	P6-7069-J	CGAGAAAGGAAGGAGTTGAAC	~2,500 bp
	P6-9543-N	GGTCATGGGCTGGGGTTTACTAT	
F11	P7-7925-J	CGCGACGGCCTTTTAAGCT	~2,000 bp
	P7-10724-N	TGAGYCGAAATCAGGTGTCTT	
F12	P8-10278-J	TCAATTTACTGATGAGGTTT	~2,300 bp
	P8-12511-N	TTGCTAACTACTTATTACCGCGGTTC	
F13	P9-12425-J	AAAACATTAGGCTGTGGCTCTA	~800 bp
	P9-13284-N	AGCGATGTGTCTGCAGTGTAGTG	
F14	P10-13065-J	TAACCTAGACCTATAGTCCGAAAA	~1,000 bp
	P10-14136-N	TCTTCTACTGGTTGGCCTCCGAT	

Table S3. List of species used in the phylogenetic analyses.

**Family**	**Genus**	**Species**	**Genome length**	**GenBank No.**
Rhacophoridae	*Polypedates*	*Polypedates mutus*	20,056 bp	MN869009
		Polypedates braueri	19,904 bp	MK687567
		Polypedates impresus	19,720 bp	MN869008
		Polypedates megacephalus FJFQ	23,798 bp	OP936085
		Polypedates megacephalus GDCH	23,848 bp	OP936086
		Polypedates megacephalus GXGL	23,996 bp	OP965715
		Polypedates megacephalus ZJJH	24,103 bp	OP965717
		Polypedates megacephalus TGPJ	23,881 bp	OP965716
		Polypedates megacephalus GDHD	15,353 bp	OP965713
		Polypedates megacephalus GDQY	17,012 bp	OP965714
		Polypedates megacephalus ZJWZ	15,361 bp	OP965718
		<u>*Polypedates megacephalus* 20130003</u>	19,952 bp	MH936677
		Polypedates megacephalus	16,473bp	AY458598
	Zhangixalus	*Zhangixalus dennysi*	18,052 bp	KM035412
		Zhangixalus omeimontis	19,604 bp	MN427892
		Zhangixalus schlegelii	21,359 bp	AB202078
		Zhangixalus dugritei	19,412 bp	MZ712011
		Zhangixalus arboreus	22,236 bp	LC565708
	Buergeria	*Buergeria buergeri*	19,959 bp	AB127977
Mantellidae	*Mantella*	*Mantella madagascariensis*	22,874 bp	AB212225
		Mantella baroni	20,945 bp	MH141579

Table S4. The partition schemes and best-fitting models selected for the 11 protein-coding genes and two rRNA genes.

Subset	Nucleotide sequence alignments	
	Subset partitions	Best model
Partition 1	*ND3*_codon1, *ND4L*_codon1, *ND1*_codon1, *ATP6*_condon1, *ND4*_codon1, *ND2*_codon1	GTR+I+G
Partition 2	*ND4L*_codon2, *ND2*_codon2, *ND4*_codon2, *ND3*_codon2, *ATP6*_codon2	TVM+I+G
Partition 3	*ATP6*_codon3, *ND4L*_codon3, *COI*_codon3, *ND3*_codon3, *COII*_codon3, *COIII*_codon3	TRN+G
Partition 4	*Cytb*_codon1, *COII*_codon1, *COI*_codon1, *COIII*_codon1	SYM+G
Partition 5	*COI*_codon2, *ND1*_codon2, *Cytb*_codon2, *COII*_codon2, *COIII*_codon2	HKY+I+G
Partition 6	*Cytb*_codon3, *ND2*_codon3, *ND4*_codon3, *ND1*_codon3	TRN+I+G
Partition 7	*ND6*_codon1, *ND6*_codon2	HKY+G
Partition 8	*ND6*_codon3	TRN+G
Partition 9	*rrnS, rrnL*	GTR+I+G

Table S5. Base compositions of five *P. megacephalus* mitogenomes.

Species name	A+T (%)				AT-skew					GC-skew				
	Mito	PCGs	rRNAs	Contral region	Mito	PCGs-H	PCGs-L	rRNAs	Control region	Mito	PCGs-H	PCGs-L	rRNAs	Control region
GXGL	61.9	59.9	59.5	68.5	-0.009	-0.056	-0.353	0.146	-0.018	-0.252	-0.293	0.464	-0.102	0.247
GDCH	61.2	59.4	59.3	67.7	-0.005	-0.050	-0.414	0.144	-0.021	-0.263	-0.299	0.525	-0.099	0.279
ZJJH	61.4	59.2	59.2	68.3	-0.003	-0.048	-0.427	0.146	-0.015	-0.267	-0.301	0.520	-0.101	0.289
FJFQ	61.1	59.2	59.0	67.8	-0.002	-0.048	-0.414	0.147	-0.015	-0.264	-0.300	0.525	-0.105	0.276
TGPJ	61.2	59.3	59.3	67.6	-0.007	-0.052	-0.392	0.143	-0.016	-0.254	-0.293	0.507	-0.089	0.262

Table S6. Locations of features in the mtDNA of *P. megacephalus* from five regions.

Table S6. 1 Location of features in the mtDNA of *P.megacephalus* ZJJH.

Feature	Strand	Position	Length (bp)	Initiation codon	Stop codon	Anticodon	Intergenic nucleotide
**$tRNA^{Thr}$**	**H**	**1-71**	71			TGT	
$tRNA^{Leu(CUN)}$	H	72-143	72			TAG	5
$tRNA^{Pro}$	L	149-217	69			TGG	1
$tRNA^{Phe}$	H	219-288	70			GAA	-2
12S rRNA	H	287-1216	930				
$tRNA^{Val}$	H	1217-1285	69			TAC	
16S rRNA	H	1286-2856	1571				2
$tRNA^{Leu(UUR)}$	H	2859-2932	74			TAA	3
ND1	H	2936-3896.	961	ATG	T(AA)		
$tRNA^{Ile}$	H	3897-3967	71			GAT	-1
$tRNA^{Gln}$	L	3967-4037	71			TTG	-1
$tRNA^{Met}$	H	4037-4105	69			CAT	
ND2	H	4106-5143	1038	ATT	TAG		-2
$tRNA^{Trp}$	H	5142-5212	71			TCA	
$tRNA^{Ala}$	L	5213-5282	70			TGC	1
$tRNA^{Asn}$	L	5284-5356	73			GTT	2
OL	L	5359-5384	26				-1
$tRNA^{Cys}$	L	5384-5448	65			GCA	
$tRNA^{Tyr}$	L	5449-5515	67			GTA	4
COI	H	5520-7073	1554	ATA	AGG		-13
$tRNA^{Ser(UCN)}$	L	7061-7131	71			TGA	1
$tRNA^{Asp}$	H	7133-7201	69			GTC	
COII	H	7202-7891	690	ATA	TAA		5
$tRNA^{Lys}$	H	7897-7966	70			TTT	

NC	H	7967-8679	713				
ATP8	H	8680-8832	153	ATG	TAG		-4
ATP6	H	8829-9507	679	ATA	T(AA)		
COIII	H	9508-10291	784	ATG	T(AA)		
$tRNA^{Gly}$	H	10292-10359	68			TCC	
ND3	H	10360-10699	340	ATG	T(AA)		
$tRNA^{Arg}$	H	10700-10768	69			TCG	
ND4L	H	10769-11053	285	ATG	TAA		-7
ND4	H	11047-12409	1363	GTG	T(AA)		
$tRNA^{His}$	H	12410-12478	69			GTG	
$tRNA^{Ser(AGY)}$	H	12479-12546	68			GCT	2
ND6	L	12549-13040	492	ATG	AGG		
$tRNA^{Glu}$	L	13041-13109	69			TTC	4
Cytb	H	13114-14269	1156	ATG	T(AA)		
CR1	H	14270-15920	1651				
ND5	H	15921-17699	1779	ATG	TAA		
CR2	H	17700-19384	1685				
ND5	H	19385-21163	1779	ATG	TAA		
CR3	H	21164-24103	2940				

”H”means gene encoded by the H-strand, “L” means gene encoded by the L-strand. Intergenic nucleotide represents noncoding base between genes. A negative number (-) denotes overlapping genes.

Table S6. 2 Location of features in the mtDNA of *P.megacephalus* FJFQ.

Feature	Strand	Position	Length (bp)	Initiation codon	Stop codon	Anticodon	Intergenic nucleotide
**$tRNA^{Thr}$**	**H**	**1-71**	71			TGT	0
$tRNA^{Leu(CUN)}$	H	72-143	72			TAG	5
$tRNA^{Pro}$	L	149-216	69			TGG	1
$tRNA^{Phe}$	H	219-288	70			GAA	-2
12S rRNA	H	287-1216	930				0
$tRNA^{Val}$	H	1217-1285	69			TAC	0
16S rRNA	H	1286-2864	1579				2
$tRNA^{Leu(UUR)}$	H	2867-2940	74			TAA	3
ND1	H	2944-3904	961	ATG	T(AA)		0
$tRNA^{Ile}$	H	3905-3975	71			GAT	-1
$tRNA^{Gln}$	L	3975-4045	71			TTG	-1
$tRNA^{Met}$	H	4045-4113	69			CAT	0
ND2	H	4114-5151	1038	ATT	TAG		-2
$tRNA^{Trp}$	H	5150-5220	71			TCA	0
$tRNA^{Ala}$	L	5221-5290	70			TGC	1
$tRNA^{Asn}$	L	5292-5364	73			GTT	2
OL	L	5367-5392	26				-1
$tRNA^{Cys}$	L	5392-5456	65			GCA	0
$tRNA^{Tyr}$	L	5457-5523	67			GTA	4
COI	H	5528-7081	1554	ATA	AGG		-13
$tRNA^{Ser(UCN)}$	L	7069-7139	71			TGA	1
$tRNA^{Asp}$	H	7141-7209	69			GTC	0
COII	H	7210-7899	690	ATA	TAA		5
$tRNA^{Lys}$	H	7905-7974	70			TTT	0
NC	H	7975-8687	713				0
ATP8	H	8688-6640	153	ATA	TAG		-4
ATP6	H	8837-9515	679	ATA	T(AA)		0
COIII	H	9516-10299	784	ATG	T(AA)		0
$tRNA^{Gly}$	H	10300-10367	68			TCC	0
ND3	H	10368-10707	340	ATG	T(AA)		0
$tRNA^{Arg}$	H	10708-10776	69			TCG	0
ND4L	H	10777-11061	285	ATG	TAA		-7
ND4	H	11055-12417	1363	GTG	T(AA)		0
$tRNA^{His}$	H	12418-12486	69			GTG	0
$tRNA^{Ser(AGY)}$	H	12487-12554	68			GCT	2

ND6	L	12557-13048	492	ATG	AGG		0
tRNA^{Glu}	L	13049-13117	69			TTC	4
Cytb	H	13122-14277	1156	ATG	T(AA)		
CR1	H	14278-15929	1652				
ND5	H	15930-17708	1779	ATG	TAA		
CR2	H	17709-19280	1572				
ND5	H	19281-21059	1779	ATG	TAA		
CR3	H	21060-23798	2739				

Notes."H"means gene encoded by the H-strand, "L" means gene encoded by the L-strand. Intergenic nucleotide represents noncoding base between genes. A negative number (-) denotes overlapping genes.

Table S6. 3 Location of features in the mtDNA of *P.megacephalus* GDCH.

**Feature**	**Strand**	**Position**	**Length (bp)**	**Initiation codon**	**Stop codon**	**Anticodon**	**Intergenic nucleotide**
**tRNA^{Thr}**	**H**	**1-71**	71			TGT	
tRNA$^{Leu(CUN)}$	H	72-142	71			TAG	5
tRNA^{Pro}	L	148-216	69			TGG	1
tRNA^{Phe}	H	218-287	70			GAA	-2
12S rRNA	H	286-1216	930				
tRNA^{Val}	H	1216-1285	70			TAC	2
16S rRNA	H	1288-2858	1571				
tRNA$^{Leu(UUR)}$	H	2859-2932	74			TAA	3
ND1	H	2936-3896.	961	ATG	T(AA)		
tRNA^{Ile}	H	3897-3967	71			GAT	-1
tRNA^{Gln}	L	3967-4037	71			TTG	-1
tRNA^{Met}	H	4037-4105	69			CAT	
ND2	H	4106-5143	1038	ATT	TAG		-2
tRNA^{Trp}	H	5142-5212	71			TCA	
tRNA^{Ala}	L	5213-5282	70			TGC	1
tRNA^{Asn}	L	5284-5356	73			GTT	2
OL	L	5359-5384	26				-1
tRNA^{Cys}	L	5384-5448	65			GCA	
tRNA^{Tyr}	L	5449-5515	67			GTA	4
COI	H	5520-7073	1554	ATA	AGG		-13
tRNA$^{Ser(UCN)}$	L	7061-7131	71			TGA	1
tRNA^{Asp}	H	7133-7201	69			GTC	
COII	H	7202-7891	690	ATA	TAA		5
tRNA^{Lys}	H	7897-7966	70			TTT	
NC	H	7967-8679	713				
ATP8	H	8680-8832	153	ATG	TAG		-4
ATP6	H	8829-9507	679	ATA	T(AA)		
COIII	H	9508-10291	784	ATG	T(AA)		
tRNA^{Gly}	H	10292-10359	68			TCC	
ND3	H	10360-10699	340	ATG	T(AA)		
tRNA^{Arg}	H	10700-10768	69			TCG	
ND4L	H	10769-11053	285	ATG	TAA		-7
ND4	H	11047-12409	1363	GTG	T(AA)		
tRNA^{His}	H	12410-12478	69			GTG	
tRNA$^{Ser(AGY)}$	H	12479-12546	68			GCT	2
ND6	L	12549-13040	492	ATG	AGG		
tRNA^{Glu}	L	13041-13109	69			TTC	4
Cytb	H	13114-14269	1156	ATG	T(AA)		
CR1	H	14270-15918	1649				
ND5	H	15919-17697	1779	ATG	TAA		
CR2	H	17698-19304	1607				
ND5	H	19305-21083	1779	ATG	TAA		
CR3	H	21084-23848	2765				

Notes."H"means gene encoded by the H-strand, "L" means gene encoded by the L-strand. Intergenic nucleotide represents noncoding base between genes. A negative number (-) denotes overlapping genes.

Table S6. 4 Location of features in the mtDNA of *P.megacephalus* TGPJ.

Feature	Strand	Position	Length (bp)	Initiation codon	Stop codon	Anticodon	Intergenic nucleotide
**tRNA^{Thr}**	**H**	**1-71**	71			TGT	0
tRNA$^{Leu(CUN)}$	H	72-143	72			TAG	5
tRNA^{Pro}	L	149-217	69			TGG	1
tRNA^{Phe}	H	219-288	70			GAA	-2
12S rRNA	H	287-1216	930				0
tRNA^{Val}	H	1217-1285	69			TAC	0
16S rRNA	H	1286-2856	1571				2
tRNA$^{Leu(UUR)}$	H	2859-2932	74			TAA	3
ND1	H	2936-3896	961	ATG	T(AA)		0
tRNA^{Ile}	H	3897-3967	71			GAT	-1
tRNA^{Gln}	L	3967-4037	71			TTG	-1
tRNA^{Met}	H	4037-4105	69			CAT	0
ND2	H	4106-5143	1038	ATT	TAG		-2
tRNA^{Trp}	H	5142-5212	71			TCA	0
tRNA^{Ala}	L	5213-5282	70			TGC	1
tRNA^{Asn}	L	5284-5356	73			GTT	2
OL	L	5359-5384	26				-1
tRNA^{Cys}	L	5384-5448	65			GCA	0
tRNA^{Tyr}	L	5449-5515	67			GTA	4
COI	H	5520-7073	1554	ATA	AGG		-13
tRNA$^{Ser(UCN)}$	L	7061-7131	71			TGA	1
tRNA^{Asp}	H	7133-7201	69			GTC	0
COII	H	7202-7891	690	ATA	TAA		5
tRNA^{Lys}	H	7897-7966	70			TTT	0
NC	H	7967-8679	713				0
ATP8	H	8680-8832	153	ATG	TAG		-4
ATP6	H	8829-9507	679	ATA	T(AA)		0
COIII	H	9508-10291	784	ATG	T(AA)		0
tRNA^{Gly}	H	10292-10359	68			TCC	0
ND3	H	10360-10699	340	ATG	T(AA)		0
tRNA^{Arg}	H	10700-10768	69			TCG	0
ND4L	H	10769-11053	285	ATG	TAA		-7
ND4	H	11047-12409	1363	GTG	T(AA)		0
tRNA^{His}	H	12410-12478	69			GTG	0
tRNA$^{Ser(AGY)}$	H	12479-12546	68			GCT	2
ND6	L	12549-13040	492	ATG	AGG		0
tRNA^{Glu}	L	13041-13109	69			TTC	4
Cytb	H	13114-14269	1156	ATG	T(AA)		0
CR1	H	14270-15908	1639				0
ND5	H	15909-17693	1785	ATG	TAA		0
CR2	H	17694-19266	1573				0
ND5	H	19267-21051	1785	ATG	TAA		0
CR3	H	21052-23881	2830				

Notes. "H"means gene encoded by the H-strand, "L" means gene encoded by the L-strand. Intergenic nucleotide represents noncoding base between genes. A negative number (-) denotes overlapping genes.

Table S6. 5 Location of features in the mtDNA of *P.megacephalus* GXGL.

Feature	Strand	Position	Length	Initiation	Stop	Anticodon	Intergenic

			(bp)	codon	codon		nucleotide
**$tRNA^{Thr}$**	**H**	**1-71**	71			TGT	
$tRNA^{Leu(CUN)}$	H	72-143	72			TAG	5
$tRNA^{Pro}$	L	149-217	69			TGG	1
$tRNA^{Phe}$	H	219-288	70			GAA	-2
12S rRNA	H	287-1216	930				
$tRNA^{Val}$	H	1217-1285	69			TAC	
16S rRNA	H	1286-2860	1575				2
$tRNA^{Leu(UUR)}$	H	2863-2936	74			TAA	3
ND1	H	2940-3900	961	ATG	T(AA)		
$tRNA^{Ile}$	H	3901-3971	71			GAT	-1
$tRNA^{Gln}$	L	3971-4041	71			TTG	-1
$tRNA^{Met}$	H	4041-4109	69			CAT	
ND2	H	4110-5147	1038	ATT	TAG		-2
$tRNA^{Trp}$	H	5146-5216	71			TCA	
$tRNA^{Ala}$	L	5217-5286	70			TGC	1
$tRNA^{Asn}$	L	5288-5360	73			GTT	2
OL	L	5363-5388	26				-1
$tRNA^{Cys}$	L	5388-5452	65			GCA	
$tRNA^{Tyr}$	L	5453-5519	67			GTA	4
COI	H	5524-7077	1554	ATA	AGG		-13
$tRNA^{Ser(UCN)}$	L	7065-7135	71			TGA	1
$tRNA^{Asp}$	H	7137-7205	69			GTC	
COII	H	7206-7895	690	ATA	TAA		5
$tRNA^{Lys}$	H	7901-7970	70			TTT	
NC	H	7971-8678	708				
ATP8	H	8679-8831	153	ATG	TAG		-4
ATP6	H	8828-9506	679	ATA	T(AA)		
COIII	H	9507-10290	784	ATG	T(AA)		
$tRNA^{Gly}$	H	10291-10358	68			TCC	
ND3	H	10359-10698	340	ATG	T(AA)		
$tRNA^{Arg}$	H	10699-10767	69			TCG	
ND4L	H	10768-11052	285	ATG	TAA		-7
ND4	H	11046-12408	1363	GTG	T(AA)		
$tRNA^{His}$	H	12409-12477	69			GTG	
$tRNA^{Ser(AGY)}$	H	12478-12545	68			GCT	2
ND6	L	12548-13039	492	ATG	AGG		
$tRNA^{Glu}$	L	13040-13108	69			TTC	4
Cytb	H	13113-14268	1156	ATG	T(AA)		
CR1	H	14269-15999	1731				
ND5	H	16000-17778	1779	ATG	TAA		
CR2	H	17779-19471	1693				
ND5	H	19472-21250	1779	ATG	TAA		
CR3	H	21251-23996	2746				

Notes. "H"means gene encoded by the H-strand, "L" means gene encoded by the L-strand. Intergenic nucleotide represents noncoding base between genes. A negative number (-) denotes overlapping genes.

Table S7. Features of *P. megacephalus* control regions.

Species	Length of CR1	Copy number of 38 bp tandem repeat in CR1	Length of CR2	Copy number of 38 bp tandem repeat in CR2	Length of CR3	Copy number of 38 bp tandem repeat in CR3	Copy number of 100 bp tandem repeats in CR3	Length of similar regions/similarity
GDCH	1649	8.6	1607	7.5	2765	7.5	11.6	99% (99.32%)
FJFQ	1652	8.6	1572	6.5	2739	9.5	10.6	99% (98.92%)
GXGL	1731	9.6	1693	8.5	2746	9.5	10.7	99% (99.76%)
ZJJH	1651	8.6	1685	9.5	2940	9.5	12.6	99%

TGPJ	1639	7.6	1573	6.5	2830	6.5	12.6	(99.45%)<br>99%<br>(98.98%)

Table S8. Pairwise genetic distance (below diagonal) and natural logarithm of geographical distance (km) (above diagonal) between geographical populations of *P. megacephalus* based on the mitogenomes. The specific collection locations of the two published sequences (MH936677 and AY458598) retrieved from the NCBI database are unknown, and geographical distances to other samples were not calculated.

**Samples**	**ZJJH**	**ZJWZ**	**GXGL**	**GDQY**	**GDCH**	**GDHD**	**FJFQ**	**TGPJ**	**MH936677**	**AY458598**
ZJJH		5.140	6.934	6.788	6.737	6.735	5.945	8.084	-	-
ZJWZ	0.002		6.988	6.792	6.734	6.701	5.649	8.081	-	-
GXGL	0.054	0.054		5.861	5.984	6.247	6.810	7.740	-	-
GDQY	0.055	0.054	0.002		4.040	5.113	6.483	7.764	-	-
GDCH	0.002	0.000	0.054	0.054		4.794	6.396	7.783	-	-
GDHD	0.055	0.054	0.001	0.002	0.054		6.311	7.792	-	-
FJFQ	0.003	0.002	0.054	0.055	0.002	0.055		7.996	-	-
TGPJ	0.039	0.039	0.057	0.058	0.038	0.058	0.039		-	-
MH936677	0.003	0.002	0.054	0.055	0.002	0.055	0.002	0.039		-
AY458598	0.055	0.055	0.001	0.003	0.055	0.002	0.055	0.058	0.056	
